# Advanced research skills: conducting literature and systematic reviews

**DOI:** 10.29173/jchla29703

**Published:** 2023-08-01

**Authors:** Eden A. Kinzel

**Affiliations:** Liaison and Education Librarian Gerstein Science Information Centre University of Toronto Toronto, Ontario, Canada

Dermody K, Farnum C, Jakubek D, Petropoulos JA, Schmidt J, Steinberg R. **Advanced research skills: conducting literature and systematic reviews [Internet]**. Toronto: Toronto Metropolitan University Library; 2022. Price: $0.00. Available from: https://pressbooks.library.torontomu.ca/graduatereviews/

Production of systematic reviews has proliferated over recent years [1]. Some librarians, including myself, might be noticing one ramification of their popularity is that more graduate students seem to be working on them, whether it’s an assignment for a class, a portion of their thesis, or as part of a job they have at their institution [2]. However, despite the increase in quantity of review articles (literature, systematic, or others), there isn’t necessarily the same increase in quality. Indeed, these publication types can often be misunderstood completely or conflated with other quite similar types of evidence synthesis. Librarians have long been involved with various stages of the evidence synthesis process and in 2022, six librarians working in Ontario published an open educational resource (OER) short course intended for graduate students entitled *Advanced research skills: conducting literature and systematic reviews* [3].

*Advanced Research Skills: Conducting Literature and Systematic Reviews* is not a typical book, but instead an online course/book hybrid. It consists of textual content, learning activities, videos, and quizzes. The course is accompanied by an online workbook (via Google Docs) that includes activities that are paired with each course module. The title is spot on when it comes to the topic – the course takes you through most of the steps of how to conduct both literature reviews and systematic reviews and is targeted towards a graduate student audience. While technically the *Cochrane handbook for systematic reviews of interventions* and the *Joanna Briggs Institute* (*JBI) manual for evidence synthesis* are designed to provide guidance specific to their respective organizations, they are looked to by most in the field as best practices in evidence syntheses [4, 5]. However, they can be quite dense and perhaps unapproachable to beginners such as graduate students. Literature providing guidance on how to conduct a literature or systematic review that is published in article, book, or course format, or authored by libraries in the form of research guides like LibGuides, tend to have some type of spin and provide guidance for reviews within a specific field or by a specific population. Six librarians comprise the authorship team of this course. According to online staff directories at the time of writing, Kelly Dermody is Head of Library Information Technology Services, Cecile Farnum and Jane Schmidt are Liaison Librarians, Dan Jakubek is the Geographic Information System (GIS) and Map Librarian, and Reece Steinberg is Head of Library Learning Services. They are all employed at Toronto Metropolitan University. The sixth author, Jo-Anne Petropoulos, is a Liaison Librarian at the Health Sciences Library at McMaster University.

This asynchronous course is organized into four modules: [I] types of reviews; [II] formulating a research question and searching for sources; [III] organizing, managing and screening sources; and [IV] strategic reading (in addition to an introduction and a conclusion/further reading section). Each module opens with distinct learning outcomes and closes with key takeaways, quizzes, and further readings. In between, you’ll find the content of the course comprised of text, examples, videos, learning activities, and directions to complete workbook activities specific to participants’ own evidence syntheses projects. The authors estimate 20 minutes to complete each module with more time for the additional resources and workbook activities and I would agree with a completion time around 1.5-2 hours for the entire course depending on reading/note-taking speed and prior familiarity with the material, again not including the workbook component. The course is discipline agnostic with examples from many disciplines, though there is an emphasis on health sciences. This OER is hosted on the PressBooks platform and despite being referred to as a course, you navigate it similarly to a book with forward and backward page buttons at the bottom of the screen. You can download an EPUB or PDF version and though some functionality such as the quizzes are lost, the links to the H5P elements are retained and displayed.

As it stands, there is no formal grading or assessment apart from the quizzes and you do not get a certificate once the course is completed. However, if the course is uploaded to a learning management system (files are provided) the quizzes can be linked to the system’s gradebook. The course is written appropriately for graduate students and defines most concepts in plain language that should be accessible to beginners even without a background in research. However, due to the lack of detail in its coverage of some stages of the review process, it may work better as an introductory exercise paired with one of the more formal and comprehensive pieces of guidance, as opposed to a standalone piece. There is an emphasis on the first few stages of the process (question formulation and searching) which makes sense as this particularly falls under the expertise of librarians, while screening and critical appraisal are relegated to a few pages each and extraction and synthesis/writing are left out completely (this is an intentional absence which is stated in the introduction). Given that there can be significant variation in critical appraisal and methods of synthesis and analysis, it would be difficult to comprehensively discuss each possibility without significantly lengthening the course.

With the authors being librarians, I would have expected a little more direction towards reaching out to a librarian for assistance with the searching portion. There were only two references to librarians in the text: the first was to their role in helping choose databases and the second was to their role on the systematic review team as the search expert.

There are some minor things that could be a bit confusing or problematic for beginners such as referring to systematic reviews as synonymous with evidence synthesis (though I recognize that some expert guidance does the same) and advising that scoping reviews take 2-3 months. I believe there is also an error in shading of Figure 2.6 in Module 2, which depicts which results would be included and excluded when using the NOT Boolean operator.

Other than its brevity forcing a few oversimplifications, I think this is a good resource for graduate students to utilize as one aspect of their literature and systematic review training. I appreciate that the authors (especially being librarians) chose to contribute to the open access movement by publishing this as an OER. The course is somewhat similar to some of the more advanced evidence syntheses LibGuides, however it is set apart by the particularly well-done learning activities and quizzes coupled with the workbook whose presence are of benefit to learners who function best with a more active role in the learning process.
